# Correlation Between Voltage and Impedance Mapping in Patients with Atrial Fibrillation

**DOI:** 10.3390/jcm14010130

**Published:** 2024-12-29

**Authors:** Antonio Taormina, Benedetta Grossi, Elisa Maria Ragaini, Giulio Falasconi, Diego Penela, Carlo Ceriotti, Luca Poggio, Paola Galimberti, Alessia Chiara Latini, Sebastiano Carli, Guido Del Monaco, Mauro Chiarito, Alessandro Sticchi, Filippo Giunti, Giulia Antonelli, Alberto Preda, Fabrizio Guarracini, Patrizio Mazzone, Gianluigi Condorelli

**Affiliations:** 1 Cardiac Arrhythmia Department, IRCCS Humanitas Research Hospital, Via Alessandro Manzoni, 6, 20089 Rozzano, Italy; diego.penelamaceda@humanitas.it (D.P.); carlo.ceriotti@humanitas.it (C.C.); paola.galimberti@humanitas.it (P.G.); alessia.latini@humanitas.it (A.C.L.); sebastiano.carli@humaniats.it (S.C.); guido.delmonaco@humanitas.it (G.D.M.); filippo.giunti@humanitas.it (F.G.); giulia.antonelli@humanitas.it (G.A.); 2 Department of Biomedical Sciences, Humanitas University, Via Rita Levi Montalcini 4, 20072 Pieve Emanuele, Italy; benedetta.grossi@polimi.it (B.G.); elisamaria.ragaini@st.hunimed.eu (E.M.R.); mauro.chiarito@humanitsa.it (M.C.); sticchialessandro@gmail.com (A.S.); gianluigi.condorelli@hunimed.eu (G.C.); 3 Cardiac Arrhythmia Department, Teknon Medical Center, 08022 Barcelona, Spain; giuliofalasconi@gmail.com; 4 Arrhythmia Unit, Ospedale Maggiore, 26900 Lodi, Italy; luca.poggio@gmail.com; 5 De Gasperis Cardio Center, Electrophysiology Unit, Niguarda Hospital, 20162 Milan, Italy; alberto.preda@ospedaleniguarda.it (A.P.); fabrizio.guarracini@ospedaleniguarda.it (F.G.); patrizio.mazzone@ospedaleniguarda.it (P.M.)

**Keywords:** paroxysmal atrial fibrillation, persistent atrial fibrillation, voltage mapping, impedance mapping, electro-anatomic map

## Abstract

**Background.** Pulmonary vein isolation (PVI) represents the cornerstone of paroxysmal (PAF) and persistent atrial fibrillation (PsAF) ablation. Impedance values provide insights on tissue conductive properties. **Methods.** Consecutive patients undergoing PAF and PsAF ablation were prospectively enrolled. All the patients underwent a preprocedural multidetector computed tomography (MDCT) to evaluate left atrial wall thickness (LAWT). Electroanatomic maps were acquired with the ablation catheter, and impedance values (Ω) and voltage amplitude (mV) of bipolar electrograms were collected. **Results.** A total of 60 patients (40 with PAF and 20 with PsAF) were included in the study. In all PAF cases, no voltage value lower than 0.5 mV was found at LA mapping; the corresponding mean impedance value was 151.5 ± 5.4 Ω. In PsAF cases, voltage values inferior to 0.05 mV have been reported in 19/20 patients. PsAF patients showed a mean impedance value of 129.1 ± 3.8 Ω. The correlation analysis between bipolar voltage and impedance reported an r_s_ value of 0.4166 (*p* < 0.001), showing a positive correlation between the two variables. On the contrary, no direct correlation was found between voltage and LAWT and between impedance and LAWT (rsv-t = 0.1838; rsi-t = 0.1133, respectively). **Conclusions.** This research study suggests a correlation between voltage amplitude and impedance values, so that impedance might be used for arrhythmogenic substrate characterization.

## 1. Introduction

Atrial fibrillation (AF) is the most prevalent cardiac arrhythmia in adults, with an estimated global impact on over 33 million individuals [1,2]. This condition presents a growing public health challenge as its incidence increases significantly with age, influenced by the aging global population and a concurrent rise in modifiable risk factors such as hypertension, obesity, and diabetes [3,4]. These conditions not only predispose individuals to the development of AF but also exacerbate its progression and complications, leading to a significant healthcare burden [5]. AF is recognized as a major contributor to increased morbidity and mortality, primarily due to its well-documented complications, which include a heightened risk of ischemic stroke, heart failure, systemic thromboembolism, and a marked reduction in quality of life [6,7,8]. Beyond these immediate health consequences, the economic implications of AF are substantial, as patients require frequent hospitalizations, long-term management strategies, and a multidisciplinary approach to care.

Atrial fibrillation is a complex arrhythmia with a progressive nature. It often begins with isolated, sporadic episodes that terminate spontaneously. Over time, these episodes tend to become more frequent and sustained, eventually evolving into permanent AF. This transition is driven by the progressive electrical and structural remodeling of the atrial myocardium, a phenomenon famously encapsulated by the phrase “AF begets AF” [9]. This concept reflects the self-perpetuating nature of the condition, where the arrhythmia itself induces changes in the atrial tissue that promote its persistence. Electrical remodeling primarily involves alterations in ion channel function and calcium handling within atrial myocytes. These changes lead to the shortening of atrial refractory periods and create a substrate conducive to reentrant circuits. Concurrently, structural remodeling involves atrial dilation and the deposition of fibrotic tissue, disrupting normal electrical conduction pathways and further facilitating the maintenance of fibrillation [10,11].

The management of AF, particularly in its persistent forms, remains a significant clinical challenge. Pulmonary vein isolation (PVI) has been a cornerstone in AF ablation strategies, with the primary goal of electrically isolating the pulmonary veins, which are key triggers for AF initiation. However, while PVI has shown excellent outcomes in patients with paroxysmal AF, its efficacy is notably reduced in persistent AF (PsAF). This disparity has prompted extensive research into adjunctive ablation techniques aimed at modifying the atrial substrate. Strategies such as targeting complex fractionated electrograms (EGMs), identifying low-voltage areas (LVAs), or addressing fibrosis-associated atrial regions have been investigated to enhance procedural success rates and improve long-term outcomes in this challenging population [12]. Nevertheless, the optimal approach for substrate modification remains an area of active debate and exploration.

Recent advancements in imaging technology have contributed significantly to the field of AF ablation. Pre-procedural imaging has gained increasing prominence as a critical tool for procedural planning and guidance. Specifically, multidetector computed tomography (MDCT) has emerged as a valuable modality for evaluating left atrial (LA) anatomy and structural characteristics. Recent studies have demonstrated the utility of MDCT-derived images in generating three-dimensional (3D) maps of LA wall thickness (LAWT). These maps can be seamlessly integrated into electroanatomic navigation systems, enabling electrophysiologists to visualize local LAWT at the catheter tip during ablation. This detailed anatomical information allows for a more tailored ablative approach, potentially enhancing both safety and efficacy [13,14,15].

Another promising parameter in AF substrate characterization is impedance. Impedance, defined as the effective resistance within an electrical circuit due to its components, provides a direct measurement of the resistive load encountered by the ablation catheter tip [16]. Studies have highlighted its reliability as an indirect marker of tissue composition, as it reflects variations in cellular and extracellular matrix properties [17,18]. However, research exploring the correlation between impedance and other substrate parameters, such as bipolar voltage and LAWT, remains limited. Understanding the interplay between these parameters could provide deeper insights into the mechanisms underlying atrial remodeling and improve ablation strategies, particularly in complex scenarios such as PsAF or post-PVI recurrences. Identifying reliable markers of arrhythmogenic substrates is essential for developing personalized, substrate-based ablation approaches that go beyond the one-size-fits-all model of PVI.

The present study seeks to address this gap by analyzing the relationship between LA impedance, LAWT, and LA bipolar voltage as measured during invasive electroanatomic mapping (EAM) procedures. By investigating these parameters, we aim to elucidate their potential interplay and evaluate their utility in guiding substrate modification during AF ablation.

## 2. Methods

### 2.1. Study Design and Enrollment

We conducted a single-center observational prospective study. Consecutive patients who underwent first-time AF ablation were prospectively enrolled between January 2022 and November 2022. All patients had documented symptomatic AF, nonresponse or intolerance to at least one (class I or III) antiarrhythmic drug, and indication for ablation in accordance with 2020 AF ESC guidelines [19]. Consistently with guidelines, we defined paroxysmal AF (PAF) in the presence of one or more documented AF episodes, all terminating spontaneously or with intervention within 7 days of onset and PsAF in the presence of at least one AF episode lasting more than 7 days [19]. Before ablation, all patients underwent MDCT to depict LA and PV anatomy, as part of our center’s usual preprocedural planning. Each patient also underwent a complete transthoracic echocardiography evaluation to assess any structural cause (i.e., valvular heart disease) of diagnosed AF. Anti-arrhythmic drugs were discontinued at least five half-lives before the procedure. Exclusion criteria were age < 18 years old, impossibility to perform a pre-procedural MDCT, previous AF ablation procedure, any clinical condition contraindicating general anesthesia, or inability to provide a signed informed consent. This study is retrospective and observational; therefore, no additional diagnostic tests or invasive procedures beyond the center’s standard clinical practice were performed to conduct the study. Essentially, only retrospective data collection was carried out. Written informed consent to collect their clinical and procedural data was obtained from all patients. This study complied with the Declaration of Helsinki [20].

### 2.2. Ablation Procedure

All procedures were carried out under general anesthesia [21,22]. Prior to catheter insertion, the groin area was sterilized, and ultrasound-guided femoral vein access was established to introduce both diagnostic and ablation catheters. In all patients, a decapolar catheter was introduced into the coronary sinus in order to have atrial and ventricular EGMs available during the entire procedure.

Transseptal puncture, a crucial step for accessing the left atrium (LA), was performed under transesophageal echocardiography guidance, utilizing a needle equipped with a pressure transducer [23,24]. This approach allowed real-time visualization, ensuring safe and accurate entry into the LA. Following successful transseptal access, unfractionated heparin (UFH) was administered to maintain an activated clotting time (ACT) exceeding 300 s, thereby reducing the risk of thromboembolic events during the intervention.

**Figure 1:**
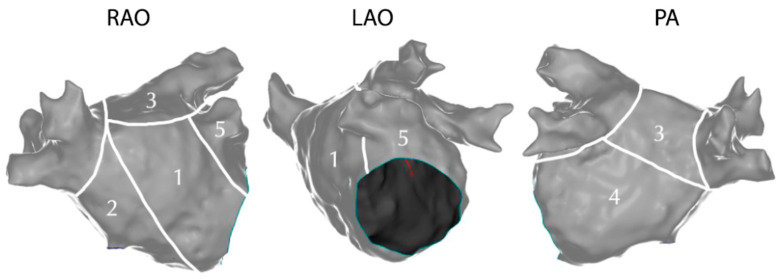
Representation of the left atrium (LA) in the standard radiological projections: Right Anterior Oblique (RAO), Left Anterior Oblique (LAO), and Posteroanterior (PA), along with its division into standard segments: anterior wall (1), septum (2), roof (3), posterior wall (4), and left atrial appendage (5). Left atrial segments: anterior wall (1), septum (2), roof (3), posterior wall (4), and left atrial appendage (5).

Procedures were performed with Stereotaxis Niobe (Stereotaxis Inc., St. Louis, MO, USA) magnetic navigation system. All patients enrolled underwent left atrium (LA) 3D sinus rhythm mapping through a Navistar RMT Thermocool ablation catheter (Biosense Webster Inc., Irvine, CA, USA) [25,26]. Magnetic torque meter values were used to ensure tip to tissue contact. Moreover, electrogram amplitude, fluoroscopy, and impedance stability were checked before acquisition. High density was ensured, with at least 1500 EGMs collected per each map, homogeneously distributed over the entire LA surface. In order to account for potential regional variability of the studied parameters, we segmented the regions of the LA as follows: (1) anterior wall; (2): septum; (3): roof; (4): posterior wall; (5) left atrial appendage (Figure 1). At least twenty EGMs per each atrial segment were collected.

PVI was performed in accordance with the CLOSE protocol [27,28]. Real-time automated display of RF applications (Visitag, Biosense Webster) was used to delineate PV encircling. Point-by-point radiofrequency (RF) applications with a maximal interlesion distance (ILD) of 6 mm and using power-controlled mode were delivered. RF delivery aimed for an ablation index (AI) target of ≥400 at the posterior/inferior wall, and ≥550 at the anterior/superior wall.

Acute PVI was confirmed by demonstrating bidirectional block: entry block was demonstrated by the absence of PV potentials inside the vein with the ablation catheter placed sequentially in each segment inside the circumferential PV line and exit block by proving absence of electric capture of the atrium during high-output pacing (10 mA at 2 ms) from inside the circumferential PV line at multiple locations. Additional “touch-up” applications were delivered at the earliest local EGM in the case of non-first pass isolation or acute PV reconnection until PVI was achieved. The procedure concluded only after confirming the absence of visual gaps between VisiTags, ensuring the continuity of the ablation lines.

Throughout the peri-procedural period, both major complications (such as death, cardiac tamponade, thromboembolism, stroke, severe pulmonary vein stenosis, permanent phrenic nerve palsy) and minor complications (including vascular-related issues not requiring intervention) were meticulously monitored and recorded to ensure patient safety and to evaluate procedural efficacy.

**EGMs analysis.** For electrogram (EGM) analysis, the acquired 3D maps underwent independent review by two senior electrophysiologists. In instances of disagreement, a third blinded reviewer was consulted to reach a consensus. Bipolar atrial EGMs, filtered within a frequency range of 30 Hz to 300 Hz, were analyzed with a focus on voltage amplitude (measured in millivolts) and corresponding impedance values (measured in ohms) at each acquisition point. Bipolar voltage amplitude was defined as the maximum peak-to-peak distance, providing insight into the electrical activity of the atrial tissue, while impedance values were determined by measuring the difference between the catheter tip and an indifferent electrode, offering information on tissue characteristics at the site of measurement.

**Multidetector computed tomography and left atrial thickness assessment.** Prior to the ablation procedure, all patients underwent MDCT. MDCT is a non-invasive imaging modality that provides high-resolution, three-dimensional images of cardiac structures, facilitating detailed evaluation of the LA and surrounding anatomy. Image acquisition was performed during an inspiratory breath-hold to minimize motion artifacts, utilizing a retrospective electrocardiogram (ECG)-gating technique. This approach synchronizes image capture with specific phases of the cardiac cycle, enhancing image clarity and accuracy. Tube current modulation was employed, adjusting the radiation dose between 50% and 100% of the cardiac cycle to optimize image quality while reducing patient exposure to radiation. To measure LAWT, the endocardial and epicardial layers of the LA were delineated using advanced image processing techniques. The endocardial layer was identified semi-automatically through threshold-based segmentation, which differentiates cardiac tissues based on their radiodensity. The epicardial layer was defined automatically, with provisions for manual adjustments to ensure precise contouring. LAWT was quantified at multiple points by calculating the perpendicular distance between corresponding endocardial and epicardial coordinates. Measurements were tailored to specific atrial segments, with MDCT slices and views selected accordingly to capture regional variations in wall thickness. At least ten measurements per atrial segment were obtained to ensure statistical robustness, and the mean value for each segment was reported. This comprehensive assessment allows for the detection of localized thickening or thinning.

**Statistical analysis.** Categorical variables are reported as percentages (%), and continuous variables are reported as mean ± standard deviation (SD). To evaluate correlations between variables of interest, Spearman rank correlation coefficients were calculated, given the non-normal distribution of certain variables, such as bipolar voltage and LAWT. This non-parametric approach is robust to deviations from normality and provides a reliable measure of the strength and direction of monotonic associations. Scatter plots were utilized to visually represent these correlations, aiding in the interpretation of statistical findings.

In addition to basic statistical tests, subgroup analyses were conducted to explore potential variations in relationships across patient groups, such as those with PAF versus PsAF. This stratification allowed for a deeper understanding of how substrate characteristics, including impedance, voltage, and LAWT, differed between these clinical phenotypes. By segmenting the left atrium into anatomical regions (anterior wall, septum, roof, posterior wall, and left atrial appendage), regional variations in these parameters were further assessed, providing insights into the spatial heterogeneity of the atrial substrate.

A level of *p* < 0.05 was considered for statistical significance. Statistical analysis was performed using R version 3.6.2 software (R Foundation for Statistical Computing, Vienna, Austria).

**Figure 2:**
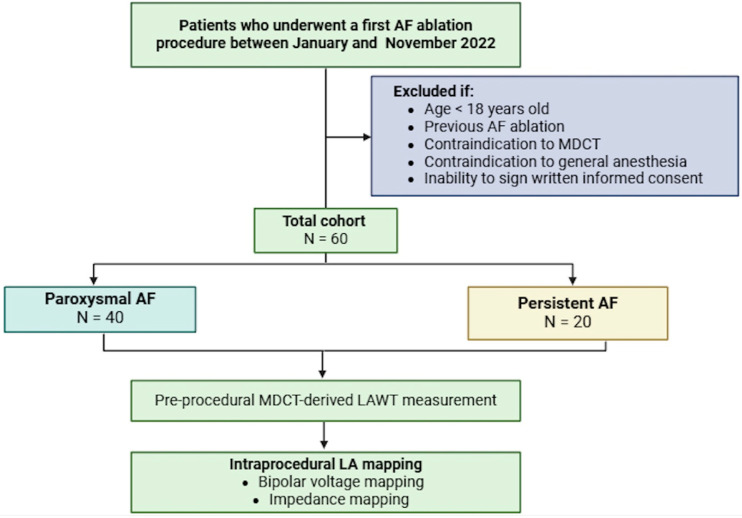
Study design flowchart.

The study design flowchart is represented in Figure 2.

## 3. Results

**Study population.** Between January and November 2022, a total of 60 patients were enrolled in the study, including 40 patients (66.6%) with PAF and 20 with PsAF (33.3%). The overall cohort consisted predominantly of male patients, with 51 men (85%), of whom 33 patients (82.5%) belonged to the PAF group and 18 patients (90%) to the PsAF group. The mean age of the study population was 58.95 ± 11.97 years, while the body mass index showed an average value of 26.88 ± 4.02 for the entire population. Hypertension was prevalent in 40% of patients, and dyslipidemia was present in 30%; diabetes mellitus affected 8.3% of patients, with a higher occurrence in the persistent AF group (15%) compared to the paroxysmal group (5%). Obstructive sleep apnea syndrome was diagnosed in 5% of patients. Heart failure diagnosis was present in 20% of patients. The mean LVEF across all patients was 52.37 ± 9.33%, while the average indexed LA volume was 36.89 ± 9.14 mL/m^2^. In the PAF group, the mean LAVi was 35.53 ± 9.50 mL/m^2^, while in the PsAF group, it was notably higher at 51 ± 6.06 mL/m^2^. This substantial increase in LAVi among PsAF patients reflects advanced atrial remodeling, which is typically observed in persistent forms of AF. No peri-procedural complications occurred. Patients’ baseline characteristics are reported in Table 1.

**Figure 3:**
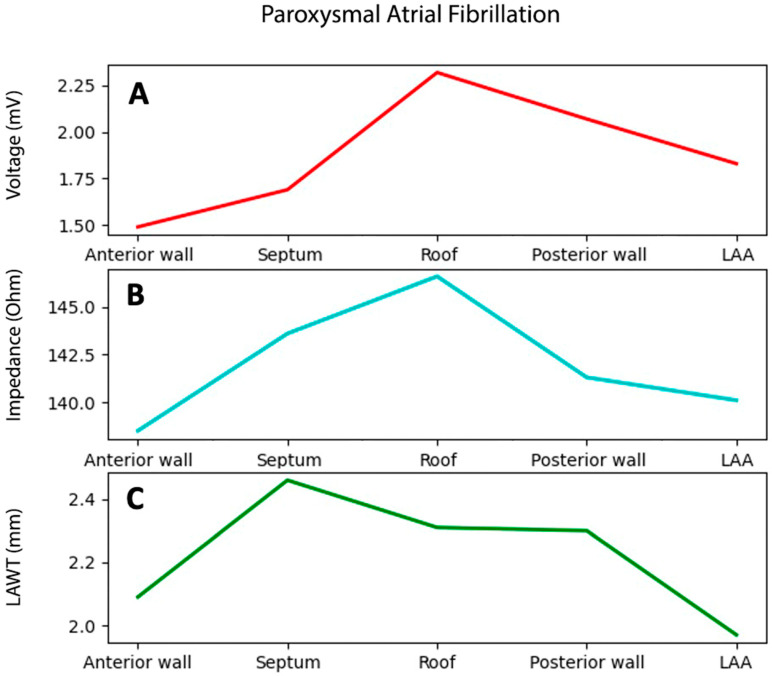
Bipolar voltage (**A**), impedance (**B**), and LAWT (**C**) trends among different atrial segments in patients with paroxysmal arial fibrillation. LAA = left atrial appendage. LAWT = left atrial wall thickness.

**Figure 4:**
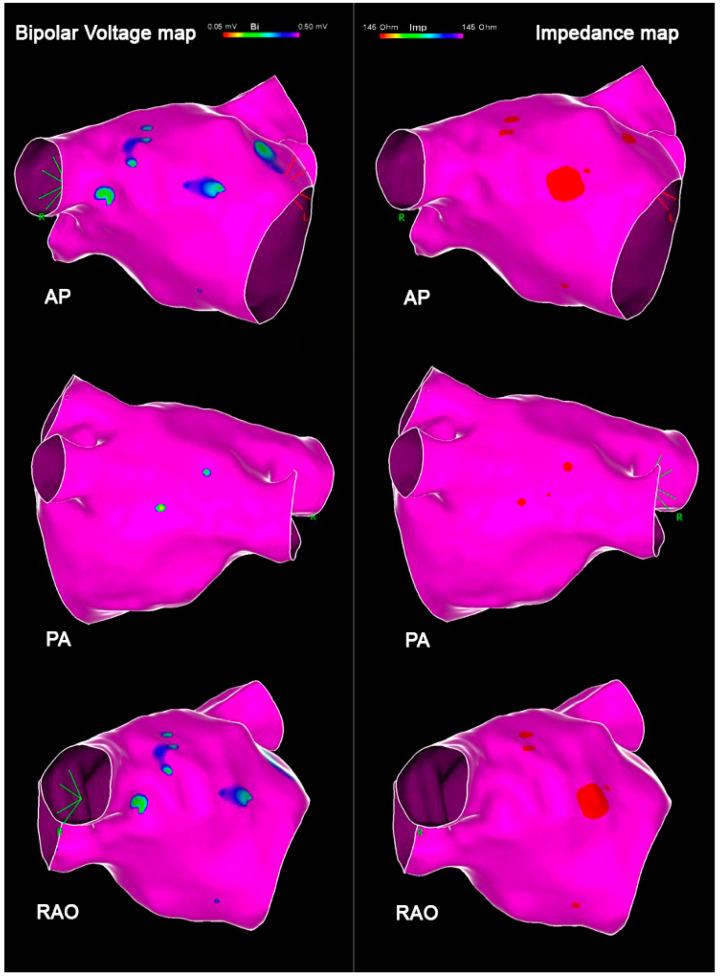
Exemplificative comparison of bipolar voltage and impedance maps in a patient with PAF: in order, anteroposterior (AP) view, posteroanterior (PA) view, and right anterior oblique (RAO) view.

**Patients with PAF.** The average number of collected EGMs per map was 1583 ± 651. In all PAF cases, no voltages lower than 0.5 mV were reported. Mean impedance value was 151.5 ± 5.4 Ω, while mean LAWT was 2.23 ± 0.36 mm. Bipolar voltage, impedance, and atrial thickness values per each atrial segment are reported in Table 2. Variable trends are shown in Figure 3 and an exemplificative comparison of voltage and impendence maps in a patient with PAF is reported in Figure 4.

**Patients with PsAF.** The average number of collected EGMs per map was 1906 ± 425. Areas with voltage values inferior to 0.05 mV were reported at the posterior wall in 19/20 patients. The average impedance value was 136.56 ± 5.9 Ω at healthy zones (voltage amplitude > 0.5 mV), while at low-voltage areas, the average impedance value dropped to 129.1 ± 3.8 Ω. Border zone areas (voltage amplitude > 0.05 mV and <0.5 mV) showed an average impedance value of 126.83 ± 3.4 Ω.

**Figure 5:**
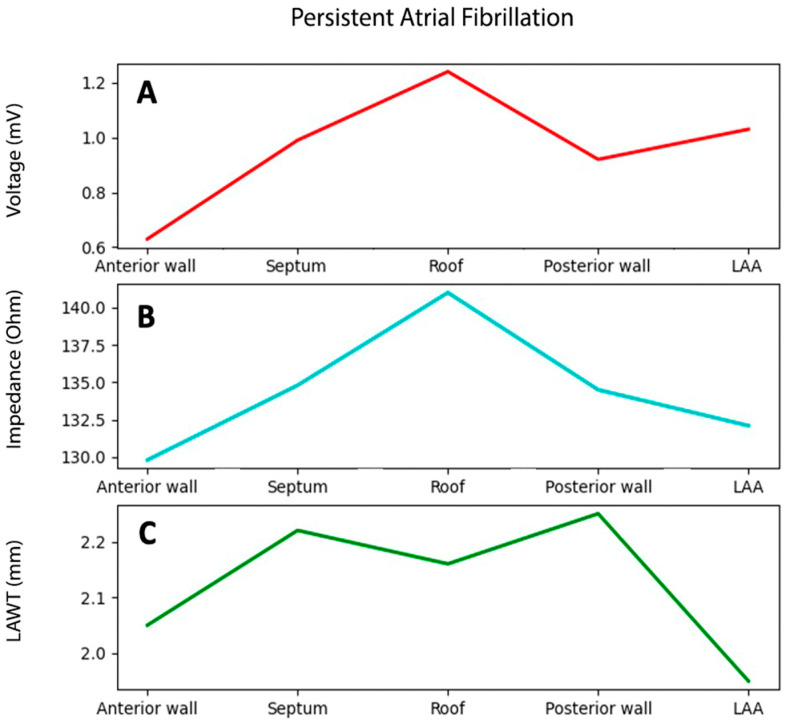
Bipolar voltage (**A**), impedance (**B**), and LAWT (**C**) trends among different atrial segments in patients with persistent arial fibrillation. LAA = left atrial appendage. LAWT = left atrial wall thickness.

**Figure 6:**
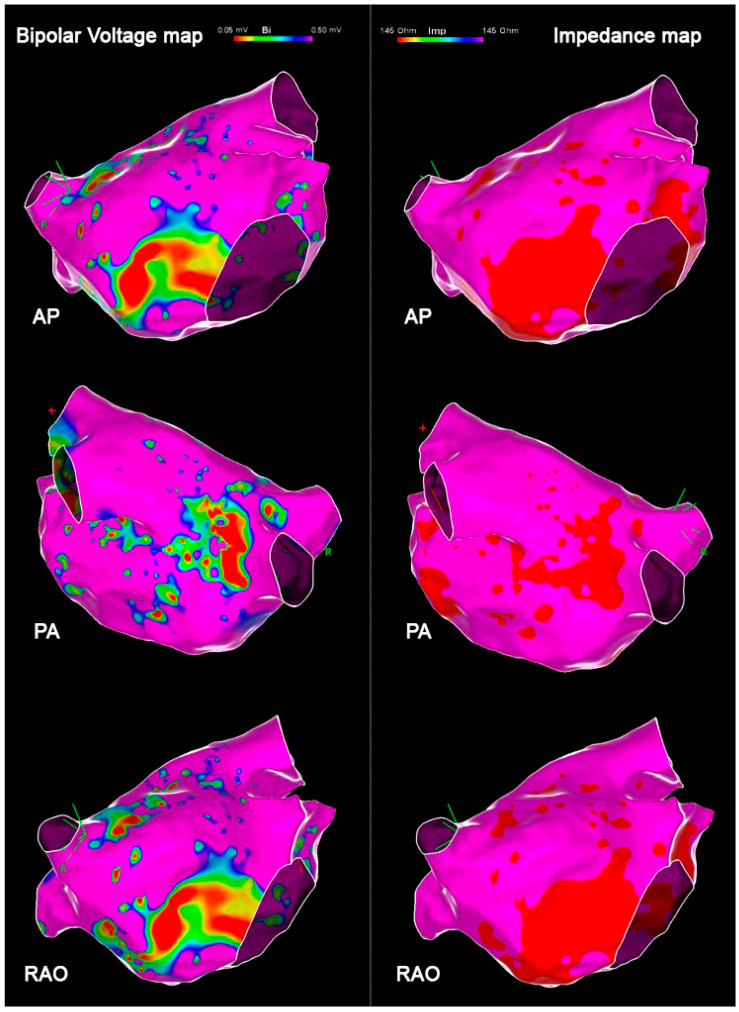
Exemplificative comparison of bipolar voltage and impedance maps in a patient with PsAF: in order, anteroposterior (AP) view, posteroanterior (PA) view, and right anterior oblique (RAO) view.

**Figure 7:**
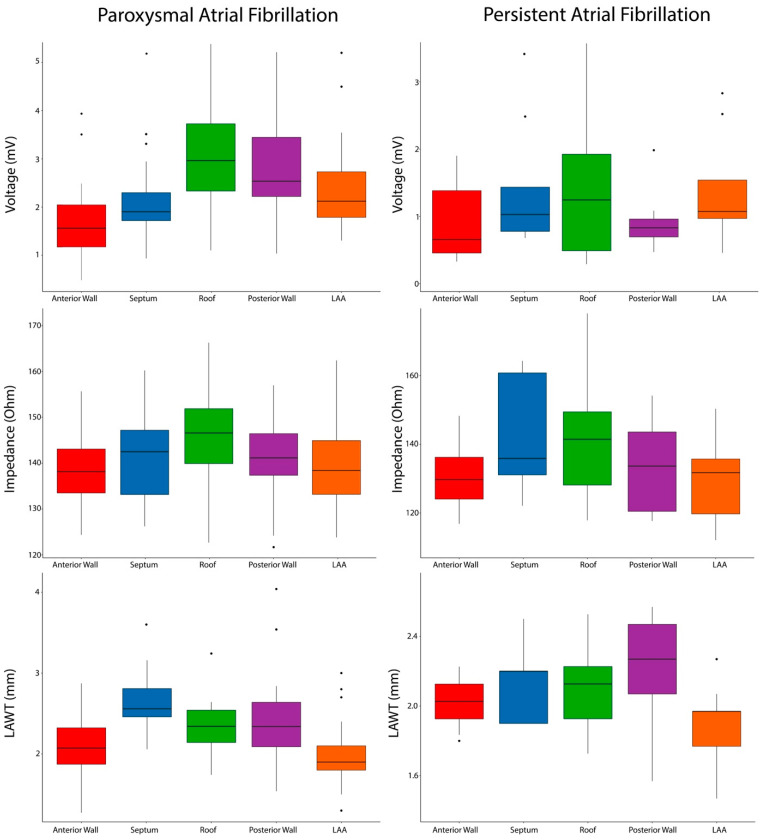
Box plots representing bipolar voltage, impedance, and LAWT, among the different LA segments of PAF and PsAF patients.

The mean atrial thickness value was 2.12 ± 0.3 mm. Bipolar voltage, impedance, and atrial thickness values per each atrial segment are reported in Table 3. Variables trends are shown in Figure 5, and an exemplificative comparison of voltage and impendence maps in a patient with PsAF is reported in Figure 6. Box plots representing bipolar voltage, impedance, and LAWT among the different LA segments of PAF and PsAF patients are reported in Figure 7.

**Figure 8:**
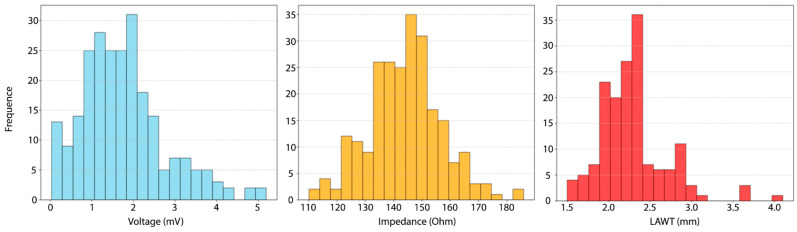
Distributions of bipolar voltage, impedance, and LAWT. The histograms illustrate that both bipolar voltage and LAWT deviate from a normal distribution, as evidenced by the Shapiro–Wilk test (*p* < 0.05). In contrast, impedance demonstrates a normal distribution.

**Correlation.** The normality of the data distribution was evaluated using the Shapiro–Wilk test. While impedance exhibited a normal distribution (*p* = 0.49), both bipolar voltage and LAWT did not (*p* < 0.05, Figure 8). Consequently, Spearman rank correlation was employed for the assessment of correlation.

**Figure 9:**
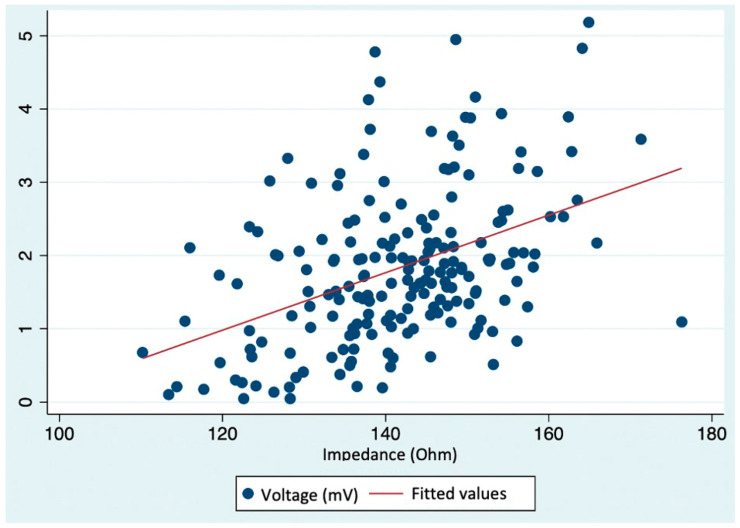
Scatter plot of bipolar voltage and impedance overlaid with a linear fit prediction plot, showing a positive moderate correlation between bipolar voltage and impedance values.

The correlation analysis between bipolar voltage and impedance values revealed a significant positive correlation, with a correlation coefficient (r_s_) of 0.4166 (*p* < 0.001), indicating a statistically significant association between the two variables. The corresponding scatter plot is depicted in Figure 9.

However, no significant correlation was observed between LAWT and bipolar voltage (*p* = 0.0912) or between LAWT and impedance values (*p* = 0.1727).

## 4. Discussion

The main findings of this research work are as follows: (i) there is a direct and statistically significant relationship between impedance and bipolar voltage values, confirmed across all atrial segments. Specifically, lower impedance values were consistently observed in regions corresponding to low voltage areas, highlighting a clear association between these two parameters; (ii) when assessing global atrial impedance values, it was found that patients with PsAF exhibited lower mean values compared to those with PAF. This distinction underscores the electrical differences in atrial substrate properties between these two clinical phenotypes of AF; (iii) no significant correlation was identified between impedance and atrial thickness, suggesting that these parameters reflect distinct aspects of atrial tissue characteristics; (iv) similarly, no correlation was observed between bipolar voltage and atrial thickness, indicating that structural parameters such as wall thickness may not directly influence voltage measurements in this context; and (v) regional analysis revealed that the highest voltage and impedance values were consistently measured in the roof segment of the atrium, whereas the lowest values were observed in the anterior wall. These findings collectively provide new insights into the spatial and electrical heterogeneity of the atrial substrate.

In the intricate exploration of AF pathophysiology, the application of advanced mapping methodologies, specifically voltage and impedance mapping, has substantially enhanced our understanding of the atrial electrophysiological environment [29]. These techniques offer complementary insights into the interplay between electrical abnormalities and structural remodeling, which are central to the perpetuation of AF. Voltage mapping, in particular, plays a pivotal role in identifying low voltage areas, which are commonly associated with fibrotic or scarred tissue. These regions are clinically significant as they represent arrhythmogenic substrates that can sustain and propagate fibrillation [30]. The ability to delineate these areas with high precision is not only crucial for guiding targeted ablation but also provides valuable insights into the underlying mechanisms of AF progression.

The observed correlation between impedance and bipolar voltage further augments our capability to identify fibrotic regions. Impedance mapping offers distinct advantages due to its sensitivity to tissue composition, particularly the extracellular matrix and fibrotic alterations. In regions of low voltage, the reduced impedance values are indicative of scarred or remodeled tissue, characterized by an increased proportion of extracellular matrix components and altered tissue hydration. This distinct relationship confers an additional layer of specificity to impedance mapping, enhancing its utility as a diagnostic and therapeutic tool. By integrating impedance data with voltage mapping, electrophysiologists can achieve a more nuanced understanding of the atrial substrate, thereby improving the precision and efficacy of substrate-based ablation strategies.

The finding of reduced mean global atrial impedance in PsAF cohorts provides a broader perspective on the electrical properties of the atrial substrate across different AF subtypes. This reduction likely reflects the extensive structural remodeling that characterizes PsAF, including advanced fibrosis and atrial dilation. Such global differences underscore the importance of tailoring ablation strategies to the specific substrate characteristics of each patient. Complementing this global perspective, the regional disparities identified in voltage and impedance profiles further highlight the spatial heterogeneity of atrial tissue. This heterogeneity is a key feature of AF pathophysiology, with certain atrial regions, such as the anterior wall, being more prone to low voltage and impedance values, while others, like the roof, exhibit higher values. Recognizing and accounting for these regional differences can refine the targeting of ablation sites and potentially improve procedural outcomes.

Despite these advancements, the biological mechanisms underlying the observed relationship between impedance and bipolar voltage remain incompletely understood. A plausible explanation lies in the unique properties of scarred tissues, which are enriched in the extracellular matrix and characterized by a higher proportion of the aqueous amorphous phase. These structural alterations likely influence both the electrical and impedance properties of the tissue. Further studies are warranted to elucidate these mechanisms, as a deeper understanding could pave the way for more effective integration of impedance mapping into routine clinical workflows.

### Clinical Implication

In the contemporary practice of AF ablation, mapping of voltage areas has become an integral and well-established technique. Voltage mapping allows electrophysiologists to identify areas of the atrium characterized by low voltage, which are often indicative of fibrotic or scarred tissue. These low-voltage zones have been increasingly recognized as critical regions in the arrhythmogenic substrate of AF, particularly in PsAF. Ablation targeting these zones has demonstrated improved procedural outcomes when compared to PVI alone, as shown in a recent randomized trial [31,32]. These findings underscore the clinical significance of incorporating voltage mapping into ablation strategies, particularly for complex cases of PsAF. However, it is important to acknowledge the limitations of voltage mapping. This technique is influenced by several factors, including catheter positioning, contact force, tissue orientation, and the effects of anesthesia, which may introduce variability in the recorded voltage values. Consequently, relying solely on bipolar voltage mapping as the definitive measure of tissue health can be suboptimal.

This study highlights the need for complementary parameters that can provide additional insights into atrial tissue characteristics. Impedance emerges as a promising candidate to fill this gap. By demonstrating a direct correlation between impedance and bipolar voltage amplitude, this research supports the hypothesis that impedance can serve as an adjunctive marker for assessing atrial substrate integrity. This is particularly relevant in situations where voltage values may be unreliable or ambiguous, such as in regions with inconsistent catheter contact or in the presence of complex scar patterns. Impedance mapping could thus offer a valuable confirmatory tool, providing electrophysiologists with greater confidence in interpreting bipolar voltage data. In clinical practice, this could translate into an additional decision-making resource for tailoring ablation strategies to the specific needs of each patient.

Prospective ablation strategies that integrate both voltage and impedance mapping hold significant potential for advancing procedural precision. By strategically targeting areas with heightened voltage and impedance values within specific atrial segments, electrophysiologists can optimize lesion placement, potentially enhancing both the efficacy and durability of ablation procedures. This approach may be particularly beneficial in addressing the spatial heterogeneity of atrial remodeling, a hallmark feature of PsAF. Furthermore, the ability to identify regions of healthy versus pathological tissue with greater accuracy could reduce unnecessary ablation in non-arrhythmogenic areas, thereby minimizing procedure times and reducing the risk of complications.

Future research should delve deeper into the temporal dynamics of impedance and voltage alterations during the progression of AF [33]. Understanding how these parameters evolve over time could pave the way for identifying predictive biomarkers of disease progression and therapeutic responsiveness. For instance, changes in impedance or voltage profiles may serve as early indicators of atrial remodeling or impending arrhythmia recurrence, enabling preemptive intervention. Moreover, longitudinal studies could clarify whether impedance mapping can reliably differentiate between reversible electrical remodeling and irreversible structural changes, further refining its clinical utility.

The integration of impedance mapping into routine clinical practice holds great promise for the era of personalized medicine. By tailoring therapeutic interventions to the unique electrophysiological and structural characteristics of each patient, clinicians may achieve significantly higher success rates in AF ablation [34]. For example, in patients with extensive atrial fibrosis, a combined approach leveraging both voltage and impedance mapping could guide substrate modification more effectively, enhancing outcomes while preserving atrial function.

In summary, the combined use of voltage and impedance mapping represents a paradigm shift in the management of atrial fibrillation. These techniques not only refine diagnostic accuracy but also provide a robust foundation for a more targeted and individualized approach to treatment. By embracing this dual-mapping strategy, clinicians can better address the complex and heterogeneous nature of AF substrates, paving the way for improved procedural outcomes and enhanced patient care.

As advancements in technology continue to accelerate, voltage and impedance mapping are poised to play a central role in shaping the future of AF research and therapeutic strategies. The adoption of these methodologies into broader clinical practice may revolutionize how electrophysiologists approach substrate characterization and ablation planning. Ultimately, this innovation has the potential to elevate patient care, reduce the burden of arrhythmia recurrence, and establish a new standard for precision medicine in the field of cardiac electrophysiology.

## 5. Limitations

This study has several limitations that should be acknowledged. First, as a single-center study, the findings may be influenced by institution-specific protocols, patient demographics, and operator expertise, potentially limiting their generalizability to other settings. Conducting multi-center studies could help validate these results across diverse populations and practices.

The small sample size represents another limitation, as it reduces the statistical power of the study and increases the likelihood of type II errors. A larger cohort would allow for more robust analyses and enable meaningful subgroup evaluations. Additionally, the lack of a control group prevents direct comparisons and limits the ability to attribute observed effects solely to the studied interventions. Including a control group would enhance the internal validity of the study and strengthen causal inferences.

Procedural variables also play a role in limiting the applicability of these findings. All procedures were performed under general anesthesia with mechanical ventilation using the Stereotaxis Niobe magnetic navigation system. These specific conditions may not reflect other procedural settings, and variations in anesthesia protocols or navigation technologies could impact electrophysiological parameters and patient outcomes. Future studies should consider the influence of different anesthetic and procedural techniques on the findings.

Discrepancies in left atrial wall thickness (LAWT) measurements compared to other studies may stem from differences in multidetector computed tomography (MDCT) acquisition protocols and segmentation tools. Standardized imaging methodologies are crucial to ensure consistency and comparability across studies.

Lastly, the observational nature of this study limits its ability to establish causation, as it can only identify associations. Prospective randomized controlled trials are needed to confirm these findings and clarify causative relationships. Additionally, the retrospective data collection may introduce biases or inaccuracies, which could be mitigated by prospective study designs to enhance data reliability. Despite these limitations, the findings provide valuable insights that can guide future research in atrial fibrillation substrate characterization and ablation strategies.

## 6. Conclusions

This research study suggests a positive correlation between impedance and bipolar voltage values across all atrial segments, with lower impedance values consistently observed in areas of reduced voltage. These results underscore the potential of impedance as a supplementary tool for characterizing arrhythmogenic substrates, particularly in patients with PsAF. Larger, multi-center studies with diverse patient populations are needed to validate these results and explore their generalizability. Additionally, prospective trials should investigate the impact of incorporating impedance mapping into ablation workflows on long-term outcomes, such as arrhythmia-free survival and procedural efficiency.

## Figures and Tables

**Table 1:** Patients’ baseline characteristics. BMI = body mass index; CAD = coronary artery disease; CKD = chronic kidney disease; DM = diabetes mellitus; HF = heart failure; LAVi = left atrial volume indexed; LVEF = left ventricular ejection fraction; PAF = paroxysmal atrial fibrillation; PsAF = persistent atrial fibrillation.

	Total (n = 60)	PAF (n = 40)	PsAF (n = 20)
Male	51 (85%)	33 (82.5%)	18 (90%)
Age	58.95 ± 11.97	59.61 ± 10.07	56.66 ± 17.63
BMI	26.88 ± 4.02	27.3 ± 3.85	25.68 ± 4.63
HF	12 (20%)	9 (22.5%)	3 (15%)
CAD	1 (1.6%)	1 (2.5%)	0 (0%)
Hypertension	24 (40%)	17 (42.5%)	7 (35%)
DM	5 (8.3%)	2 (5%)	3 (15%)
CKD (eGFR < 60 mL/min/1.73 m^2^)	0 (0%)	0 (0%)	0 (0%)
OSAS	3	1	2
Dyslipidemia	18 (30%)	12 (30%)	6 (30%)
LVEF, %	52.37 ± 9.33	51.68 ± 9.80	54.78 ± 7.51
LAVi, mL/m^2^	36.89 ± 9.14	35.53 ± 9.50	51 ± 6.06
Amiodarone assumption	11 (18.3%)	9 (22.5%)	2 (10%)
Class IC antiarrhythmic assumption	12 (20%)	10 (25%)	2 (10%)

**Table 2:** Bipolar voltage, impedance, and left atrial wall thickness of PAF patients, according to the LA segments. Values are reported as mean ± SD. LA = left atrium; LAA = left atrial appendage; PsAF = persistent atrial fibrillation.

	LA Segments of Patients with Paroxysmal Atrial Fibrillation				
	Anterior Wall (n = 40)	Septum (n = 40)	Roof (n = 40)	Posterior Wall (n = 40)	LAA (n = 40)
**Voltage (mV)**	1.49 ± 0.88	1.69 ± 0.86	2.32 ± 1.21	2.07 ± 1.21	1.83 ± 1.12
**Impedance (Ohm)**	138.5 ± 7.2	143.6 ± 7.5	146.6 ± 8.7	141.3 ± 5.6	140.1 ± 6.9
**Atrial Thickness (mm)**	2.09 ± 0.27	2.46 ± 0.49	2.31 ± 0.35	2.30 ± 0.37	1.97 ± 0.34

**Table 3:** Bipolar voltage, impedance, and left atrial wall thickness of PsAF patients, according to the LA segments. Values are reported as mean ± SD. LA = left atrium; LAA = left atrial appendage; PsAF = persistent atrial fibrillation.

	LA Segments of Patients with Persistent Atrial Fibrillation				
	Anterior Wall (n = 20)	Septum (n = 20)	Roof (n = 20)	Posterior Wall (n = 20)	LAA (n = 20)
**Voltage (mV)**	0.63 ± 0.39	0.99 ± 0.55	1.24 ± 0.64	0.92 ± 0.55	1.03 ± 0.75
**Impedance (Ohm)**	129.8 ± 6.1	134.8 ± 7.7	141.0 ± 9.2	134.5 ± 4.9	132.1 ± 7.0
**Left Atrial Wall Thickness (mm)**	2.05 ± 0.30	2.22 ± 0.28	2.16 ± 0.23	2.25 ± 0.35	1.95 ± 0.36

## Data Availability

The data that support the findings of this study are available from the corresponding author, upon reasonable request.

## Abbreviations and Acronyms

ACT	activated clotting time
AI	ablation index
AP	anteroposterior
AF	atrial fibrillation
BMI	body mass index
CAD	coronary artery disease
EAM	electroanatomic mapping
EGM	electrogram
ILD	interlesion distance
LA	left atrium
LAWT	left atrial wall thickness
LVA	low-voltage area
MDCT	multidetector computer tomography
PA	posteroanterior
PAF	paroxysmal atrial fibrillation
PsAF	persistent atrial fibrillation
PV	pulmonary vein
PVI	pulmonary vein isolation
RAO	right anterior oblique
RF	radiofrequency
SD	standard deviation
SR	sinus rhythm
HF	heart failure
DM	diabetes mellitus
IHD	ischemic heart disease
CKD	chronic kidney disease
LAVi	left atrial volume indexed
LAA	left atrial appendage
UFH	unfractioned heparin
